# Strategic career behaviours among hybrid workers: testing a general European model

**DOI:** 10.3389/fpsyg.2024.1347352

**Published:** 2024-04-02

**Authors:** Kiall Hildred, Margarida Piteira, Sara Cervai, Joana Carneiro Pinto

**Affiliations:** ^1^Universidade Católica Portuguesa, Faculdade de Ciências Humanas, CRC-W, Lisbon, Portugal; ^2^ISCAL, Lisbon, Portugal; ^3^University of Trieste, Trieste, Italy

**Keywords:** strategic career behaviours, hybrid working, career management, European workers, antecedents and consequences

## Abstract

**Introduction:**

This study investigates the antecedents and consequences of strategic career management behaviours in a sample.

**Methods:**

A total of 739 employees (Male = 442, 59.8%) with a mean age of 27.64 years (SD = 8.48; Range = [18, 70]), working mostly full-time (*n* = 398, 53.9%) and with 46.35% of their work being done hybrid-like participated in this study. The study tested perceived self-efficacy, desire for career control and perceived organizational support as predictors of strategic career behaviours. And tested strategic career behaviours as predictors of perceived career control, objective and subjective career success, and career satisfaction.

**Results:**

Results indicate objective career success was not related to the antecedent variables of strategic career behaviours and hence was removed from the model. Regression and mediation analyses demonstrated that perceived self-efficacy and desire for career control are good predictors of the use of strategic career behaviours, but perceived organizational support is not; strategic career behaviours are reasonable predictors of perceived control, and very strong predictors of subjective career success and career satisfaction.

**Discussion:**

Strategic Career Behaviours were found to play only a partial mediating role in the present model suggesting that further analysis is required to determine whether they play a central role in the relationships between the antecedents and consequences in the present model, or whether they should be considered a contributing but merely parallel factor. These results will support career management programs, accounting for idiosyncrasies of hybrid work.

## Introduction

The social-distancing and isolation measures put in place in response to the COVID-19 pandemic led many to adopt or require flexible working arrangements (Milasi et al., 2021). It is reasonable to expect that this shift affected not just where people conduct their work, but also how they think about and manage their career goals, their progress and their aspirations.

This shift also posed many challenges for individual career management, as it has been found that many employees feel that hybrid and remote work negatively affects their long-term career prospects and opportunities for career advancement (Lott and Abendroth, 2020; Tavares et al., 2020). This concern has been reified by work (e.g., Bloom et al., 2015) that suggests that these working arrangements reduce the likelihood of career advancement because it clashes with the belief that the measure of one’s productivity depends on the time spent on the job (as per Possenriede et al., 2014; Green et al., 2020). This also seems to spill over into the perception office-bound workers and managers have of their hybrid-and remote-working colleagues, affecting the consistency of the latter workers’ performance (Baruch, 2000). Furthermore, without direct communication and social interaction with colleagues and superiors, these workers may also worry that they are missing out on opportunities for mentorship and coaching, as well as opportunities to develop a corporate identity with the company they work for (De Vries et al., 2019; Tavares et al., 2020). Also, the autonomy implied or expected from them lumps them with greater responsibility for defining their roles and for managing their long-term career trajectory (Wrzesniewski and Dutton, 2001; Raabe et al., 2007). Hence, it is of particular importance to understand the specific challenges faced by those in hybrid and remote work (Satici et al., 2020), especially given that the ubiquity of this form of work may become a permanent feature of jobs in the future.

The main contribution of this study lies in the expansion of research on the interaction between hybrid and remote work with career behaviours, antecedents, and outcomes within the European context. While previous research has focused on pairwise relationships between these factors (e.g., King, 2000, 2004; Lent and Brown, 2006, 2013; Lent et al., 2020), the study aims to provide a comprehensive understanding of how strategic career behaviours are influenced by the dynamics of hybrid workers across a diverse European population.

By extending the work of Sotto-Mayor et al. (2023) to include a broader European sample (17 countries), the study addresses the limitations of previous research, which was confined to Spain and Portugal. This expansion allows for a more robust examination of the antecedents and consequences of strategic career behaviours among remote workers, considering variations in work dynamics, cultural contexts, and organizational structures across different European countries.

Furthermore, the study acknowledges the potential negative impacts that remote work may have on individuals’ ability to manage their career goals and other factors included in the model. By investigating these interactions within a larger framework, the study contributes valuable insights into the complexities of hybrid and remote work and its implications for career development and organizational outcomes in diverse European contexts.

In summary, the main contribution of the study lies in its effort to advance our understanding of strategic career behaviours among remote workers in Europe, offering insights that can inform organizational policies, practices, and interventions aimed at supporting employees in navigating the challenges and opportunities associated with hybrid and remote work arrangements.

The present work used the Kaleidoscope Career Model (KCM; Mainiero and Sullivan, 2005; Sullivan and Mainiero, 2008) as the central point for a broader model of the antecedents and consequences of strategic career behaviours. The KCM is composed of three strategic career behaviours, named authenticity, balance and challenge. Authenticity refers to moving one’s career to be in alignment with one’s personal values. Balance refers to satisfactorily allocating one’s time and energy between one’s career and non-career duties. Challenge refers to seeking out challenges in one’s work in order to increase opportunities for growth and career advancement. The KCM hypothesis is that workers focus in varying degrees on these three strategies at different times in their life or career and as their goals change. There is not a very extensive literature on the KCM, considering that it is a relatively recent concept, but from what we could investigate, there are no studies that have analysed these behaviours among remote workers.

In this study, the strategic career behaviours are treated as a single variable, based on the conception of some authors regarding these behaviours as a collective and interrelated set of actions that individuals undertake to strategically advance their careers (e.g., Sullivan and Mainiero, 2008; Simmons et al., 2022), allowing them to be aggregated into a composite variable. The decision to treat them as a single variable is also motivated by the need to simplify the model for analytical purposes and to capture the overall impact of strategic career behaviours on the outcome of interest.

**Figure 1:**
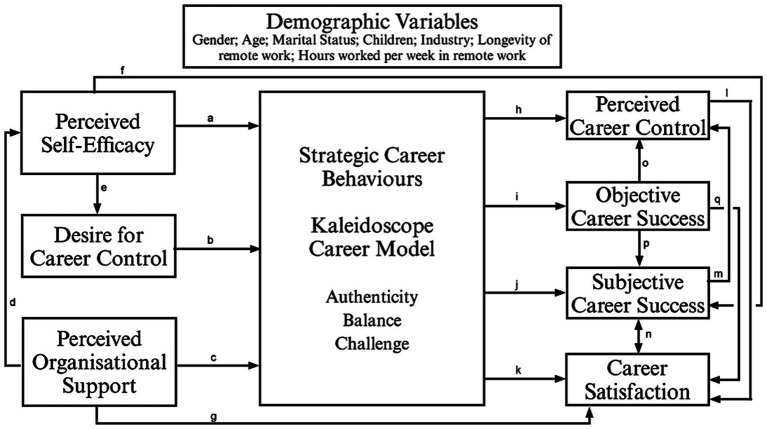
Conceptual model using the Kaleidoscope career model and its antecedents and consequences on remote workers.

The antecedents of Strategic Career Behaviours chosen for the broader model (Figure 1) were Perceived Self-Efficacy (PSE), Desire for Career Control (DCC) and Perceived Organizational Support (POS), and the consequences were Perceived Career Control, (PCC) Objective Career Success (OCS), Subjective Career Success (SCS) and Career Satisfaction (SAT).

The selection of variables or components in the broader model of strategic career behaviours (Figure 1) represents a thorough understanding of the elements that influence individuals’ career trajectories and outcomes in remote work situations.

- Perceived self-efficacy (PSE) is an individual’s conviction in their ability to successfully complete tasks and achieve goals. High levels of perceived self-efficacy are likely to promote proactive career management, goal setting, and challenge-facing resilience in the context of strategic professional activities (e.g., Judge and Bono, 2001; Lent and Brown, 2013; Li et al., 2019; Lent et al., 2020);
- Desire for career control (DCC) refers to people’s desire to actively direct and influence their career pathways. This variable measures how much people value flexibility, autonomy, and decision-making power in their professional endeavours. These factors might have an impact on people’s strategic career behaviours and results (e.g., King, 2000, 2004);
- Perceived organizational support (POS) refers to how employees feel about the degree to which their employer appreciates their work, is concerned about their welfare, and supports their professional growth. High perceived support organisations are likely to encourage higher levels of employee engagement, dedication, and investment in strategic career behaviours (e.g., Eisenberger et al., 2016; Rhoades et al., 2020);
- Perceived career control (PCC) refers to how people believe they can steer and affect their professional pathways. It includes sentiments of empowerment, independence, and self-assurance in handling professional choices and changes, all of which can affect one’s level of job satisfaction and success (e.g., King, 2000, 2004);
- Objective career success (OCS) describes observable indicators of professional accomplishment and advancement, like employment stability, pay raises, and promotions. This variable offers an external indicator of career accomplishment and advancement, which can be impacted by corporate support as well as people’s strategic professional behaviours (e.g., Ng et al., 2005; De Vos and Soens, 2008; Hildred et al., 2023);
- Subjective career success (SCS) refers to how people subjectively assess their work achievements, level of fulfilment, and compatibility with their personal beliefs and aspirations. It represents the extent to which people believe they are successful and content in their jobs, and this perception can be impacted by a number of variables, including self-efficacy, career control, and organizational support (e.g., Ng et al., 2005; Lent and Brown, 2013; Hildred et al., 2023);
- Career satisfaction (SAT): measures how happy and fulfilled people are in their careers overall. It includes a range of elements including work-life balance, career fulfilment, job satisfaction, and alignment with both personal and professional goals. A mix of internal (such as self-efficacy and a need for control) and external (such as organizational support and career success) elements affect career happiness (e.g., Nikandrou and Galanaki, 2016).

In conclusion, these factors were selected in order to better understand the causes and effects of strategic career actions among remote workers, both theoretically and empirically. They offer a thorough framework for analysing the contextual, organizational, and human elements that influence career development and results in remote work environments (Kossek et al., 1998; King, 2000; Lau and Pang, 2000; Desrosiers, 2001; Sturges et al., 2002; Lent and Brown, 2006; Raabe et al., 2007).

The next sections will detail the relationships between antecedents, strategic career behaviours, and consequences, theoretically supporting the development of the model we intend to test.

## Relationships between antecedents and strategic career behaviours

Self-efficacy has been associated with career behaviour as far back as the study by Lent et al. (1987). Later work by Raghuram et al. (2003) showed self-efficacy to be positively related to the career management behaviour of remote workers. However, the prior study using the present model by Sotto-Mayor et al. (2023) supported a predictive relationship between perceived self-efficacy and strategic career behaviours, but in the negative direction.

A potential explanation for this inverse relationship is that those who see themselves as highly-efficacious may not feel any subsequent need to engage in strategic career behaviours to improve this perception, where as those with low perceived self-efficacy seek to engage in behaviours that will make them feel more efficacious.

The conflict between the studies above may be due to two main reasons. First, while they both include samples of remote workers, the prior study by Sotto-Mayor et al. (2023) focused on a European sample, whereas Raghuram et al. (2003) was conducted with U.S. sample, and second, there may be differences in outcomes based on the subtle differences in the measures used for self-efficacy and perceived self-efficacy [Self-efficacy, 3 items, taken from Sherer et al. (1982) in the case of Raghuram et al. (2003); Perceived Self-efficacy, 11 items, taken from Whitely et al. (1991) in the case of Sotto-Mayor et al. (2023)].

The prior study by Sotto-Mayor et al. (2023) also found that perceived self-efficacy correlated negatively with desire for career control and was a significant predictor of it, which suggest that having low perceived self-efficacy leads to a stronger desire for control over one’s career. Kuijpers and Scheerens (2006) have argued that career control and organisational support are important components in career self-management. In the current working climate, with its increase in remote working and the isolation that comes with it, having control over one’s career and being supported in self-management are expected to be ever more important for engaging in strategic career behaviours.

Indeed, the prior study by Sotto-Mayor et al. (2023) showed that desire for career control positively correlated with strategic career behaviours, suggesting that desiring career control leads to positive actions to gain that control. This aligns with King (2004) who discussed an association between perceived self-efficacy and career control but suggested that the relationship from career control to self-efficacy would be positive, as the desire for career control leads to good outcomes, this has a positive effect on perceived self-efficacy. This suggests a more dynamic, non-linear relationship between these two factors.

While Nabi (2000) has argued that the motivation to engage in strategic career behaviours is directly influenced by organisational support, the prior study by Sotto-Mayor et al. (2023) showed no direct relationship between perceived organizational support and strategic career behaviours. However, it did show that perceived organizational support was a significant positive predictor of perceived self-efficacy (in addition to desire for career control), suggesting that being supported by one’s organisation improve one’s belief in their abilities, and that perceive organisational support may only affect strategic career behaviours indirectly through perceived self-efficacy and desire for career control.

As the present study follows from Sotto-Mayor et al. (2023), using the same model in a European sample, we propose the following hypotheses:

*Hypothesis 1a*: Perceived self-efficacy negatively predicts strategic career behaviours (path a).

*Hypothesis 1b*: Desire for career control positively predicts strategic career behaviours (path b).

*Hypothesis 1c*: Perceived organisational support has only an indirect relationship to strategic career behaviours (path c).

*Hypothesis 1d*: Perceived organisational support positively predicts perceived self-efficacy (path d).

*Hypothesis 1e*: Perceived self-efficacy negatively predicts desire for career control (path e).

## Relationships between strategic career behaviours and consequences

The work by Koekemoer and Crafford (2019) would suggest positive links between factors in the Kaleidoscope Career Model and the measures of subjective and objective career success, and the work by Stumpf and Tymon (2012) showed strong links between objective and subjective career success, while several studies show at least a weak to moderate link between the two variables (e.g., Dette et al., 2004; Arthur et al., 2005; Hall and Chandler, 2005; Ng et al., 2005). There are many possible reasons for an interaction between these two variables. A higher salary may lead to more freedom to engage in behaviours that boost subjective career success, or it could be the result of more stress and hence contribute to lowering subjective career success. These relationships may also change over time as people adapt to their base level of salary, position or lifestyle.

Malik et al. (2019) showed that work-life balance positively predicted subjective career satisfaction, and Najam et al. (2020), showed work-life balance to moderate the effect of career commitment on subjective career success.

Furthermore, Qiu and Dauth (2022) found that work-family balance was an important mediator in the relationship between the effects of telework intensity and job satisfaction, suggesting that the strategic career behaviour of balance predicts job satisfaction. Both Raabe et al. (2007) and Lau and Pang (2000) showed positive connections between strategic career behaviours and career satisfaction, while Greenhaus et al. (1990) found a negative relationship between the two. In relation to the other paths in the model, the work by Sönmez et al. (2021) showed a positive effect of subjective career success on job satisfaction.

Despite these mixed findings, the prior study by Sotto-Mayor et al. (2023) showed no correlations between strategic career behaviours and any of the consequent factors in the model (perceived career control, objective career success, subjective career success, and career satisfaction). However, the study did show that subjective career success was positively correlated with both objective career success and career satisfaction. Regarding the conflict with those studies focused on job satisfaction and the lack of relationships found in Sotto-Mayor et al. (2023), this is possibly due to slight differences in the measures of *career* satisfaction vs. *job* satisfaction, as the former focuses on the over-arching course of a career and not on the current and specific job one is in. However, Lau and Pang (2000) have argued that, at least in the early stages of one’s career, these terms are indistinguishable.

The work by Simmons et al. (2022) used career satisfaction as a variable, and the work gives a wide and direct investigation into the relationships between the Kaleidoscope Career Model and other career outcomes. They looked at the effects of the individual components of the model (authenticity, balance and challenge) and their findings suggest that: while the level of emphasis on authenticity at a given time has no effect on career outcomes, increases (or decreases) in this emphasis over time lead to decreases (or increases) in career satisfaction; an emphasis on balance *negatively* predicted salary, but did not predict promotions, career satisfaction, or promotion rate, except that changes in the emphasis on balance over time did positively predict promotion rate; and an emphasis on challenge at any given moment did not predict any of the career outcomes, but changes in this emphasis over time positively predicted all promotions, promotion rate, salary and career satisfaction. Additionally, in the study developed by Guest and Rodrigues (2015), the authors found that perceived career control is a relevant variable in explaining job, career and overall life satisfaction.

But very few of the studies above were conducted on broad European samples of workers, or on remote workers. As the present study follows from Sotto-Mayor et al. (2023), using the same model in a larger European sample that includes remote workers, it is valuable to reconsider the expected relationships in the model developed in the prior study.

Hence, drawing on previous findings and on the prior study, we propose the following hypotheses:

*Hypothesis 2a*: Treated as a single variable, strategic career behaviours positively predict perceived career control, objective career success, subjective career success and career satisfaction (paths h–k).

*Hypothesis 2b*: Perceived career control positively predicts career satisfaction (path l).

*Hypothesis 2c*: Objective career success positively predicts perceived career control, subjective career success and career satisfaction (paths o–q).

*Hypothesis 2d*: Subjective career success positively predicts perceived career control and career satisfaction (path m and n).

## Relationships between antecedents and consequences

Regarding relationships between the antecedents and consequences, various results have been found across studies. Abele and Spurk (2009) found that self-efficacy had a positive impact on measures of objective career success and career satisfaction. This latter relationship was also found by Rigotti et al. (2020), and a positive correlation between self-efficacy and career success (mixed objective and subjective measures) was found by Wu et al. (2022). Furthermore, both Badri et al. (2013) and Ballout (2009) found self-efficacy to play an important positive role in the relationship of objective and subjective career success (satisfaction) to other variables.

Regarding the role of perceived organisational support in career outcomes, a study by Agrawal and Singh (2022) found that perceived organizational support moderated the relationship between career behaviours and subjective career success; Armstrong-Stassen and Ursel (2009) found career satisfaction to act as a mediator between perceived organizational support and various strategic career behaviours; and Dose et al. (2019) found indirect positive links between perceived organisational support and objective and subject career success.

The prior study by Sotto-Mayor et al. (2023) also found self-efficacy to predict subjective career success and career satisfaction, but not objective career success or perceived career control. Perceived organisational support positively predicted career satisfaction, and was negatively correlated with objective career success, but did not correlate with subjective career success. Also, the study found no correlations between desire for career control and any of the consequent variables.

Again, many of the above-cited studies were conducted on non-European samples with workers in traditional (non-telework) settings [e.g., Badri et al., 2013 used a sample from United Arab Emirates; Ballout, 2009 used a sample from Lebanon; Wu et al., 2022 used a sample from China]. Hence, it is important to reconsider these relationships in the present European sample including teleworkers. In light of the above findings, we propose the following hypotheses:

*Hypothesis 3a*: Perceived self-efficacy positively predicts objective career success, subjective career success (path f) and career satisfaction.

*Hypothesis 3b*: Desire for career control has no direct effect on perceived career control, objective career success, subjective career success or career satisfaction.

*Hypothesis 3c*: Perceived organizational support positively predicts objective career success, subjective career success and career satisfaction (path g).

## Strategic career behaviours as a mediator of the relationships between antecedents and consequences

The prior work by Sotto-Mayor et al. (2023) found no evidence to suggest Strategic Career Behaviours as a mediator between the antecedent and consequent variables. However, only the relationship between perceived self-efficacy and career satisfaction was tested. Therefore, it is worthwhile to reconsider these relationships and the mediating effects of Strategic Career Behaviours within a larger and more diverse sample.

*Hypothesis 4*: Strategic career behaviours mediate the effects of perceived self-efficacy, desire for career control and perceived organizational support on perceived career control, objective career success, subjective career success and career satisfaction.

## Method

### Participants

The respondents comprised a total of 739 (Male = 442, 59.8%) European individuals with a mean age of 27.64 years (SD = 8.48; Range = [18, 70]), mostly single (*N* = 499, 67.5%) with no children (639, 86.5%), working mostly full-time (*N* = 398, 53.9%) and with 46.35% of their work being done remotely. The countries represented in the sample were Portugal (*n* = 236, 31.9%), Poland (*n* = 192, 26.0%), Italy (*n* = 80, 10.8%), Spain (*n* = 65, 8.8%), Greece (*n* = 50, 6.8%), the United Kingdom (*n* = 35, 4.7%), Hungary (*n* = 32, 4.3%), Czech Republic (*n* = 10, 1.4%), Estonia (*n* = 8, 1.1%), Finland (*n* = 6, 0.8%), Germany (*n* = 6, 0.8%), Belgium (*n* = 4, 0.5%), France (*n* = 4, 0.5%), Austria (*n* = 3, 0.4%), Denmark (*n* = 3, 0.4%), Sweden (*n* = 3, 0.4%), and Switzerland (*n* = 2, 0.3%).

Most worked in small (1–25 employees: *N* = 250, 33.8%; <250 employees: *N* = 204, 27.6%), private organisations (*N* = 550, 74.4%), and mostly in the industries of: media, cultural, graphical (*N* = 108, 14.6%); mechanical and electrical engineering (*N* = 82, 11.1%); commerce (*N* = 80, 10.8%); education (*N* = 71, 9.6%); health care and social assistance services (*N* = 71, 9.6%); and financial services (*N* = 64, 8.7%).

Most were earning low salaries (<1,000€: 42.2% [*N* = 312]; 1,000–1,499€: 28.6% [*N* = 211]), had received on average 0.94 (SD = 1.263, [0, 10], *N* = 738) promotions over the previous 6 years period and a 13.549% (SD = 26.414%, [−100, 100], *N* = 707) increase in their salaries.

The levels of education attained across the participants showed that 0.1% (*N* = 1) and 37.1% (*N* = 274) had complete primary and secondary education, respectively; while 40.7% (*N* = 301), 20.8% (*N* = 154), and 1.2% (*N* = 9) had completed a bachelor’s, master’s or PhD, respectively.

### Instruments

The online questionnaire^1^ included sections on: personal variables, such as age and gender; sociodemographic variables, including country of residence and family variables; employment status, including measures of salary change (%SI) and promotions (PRO) for use as measures of Objective Career Success; Strategic Career Behaviours using the Kaleidoscope Career Model; the antecedents, Perceived Self-Efficacy, Desire for Career Control, and Perceived Organisational Support; and the consequences, Perceived Career Control, Subjective Career Success, Career Satisfaction.

The variables used in the model are detailed below (see also Table 1).

**Table 1:** Psychometric data and reliability of instruments used in the model.

Instruments	Mean	SD	Median	Mode	Min, Max	Items	Cronbach’s *α*
Perceived Self-Efficacy Whitely et al. (1991)	39.80	6.282	40	42	19, 54	11	0.787
Desire for Career Control King (2000)	26.72	3.20	27	28	15, 35	7	0.552
Perceived Organizational Support Eisenberger et al. (1986)	34.95	5.09	36	36	20, 49	11	0.669
Strategic Career Behaviours Kaleidoscope Career Model, Sullivan and Mainiero (2008)	50.68	9.24	51	51	20, 73	15	0.831
Perceived Career Control Kuijpers and Scheerens (2006)	17.35	3.95	18	19	5, 25	5	0.862
Subjective Career Success Briscoe et al. (2021), Importance	78.21	9.80	79	78	38, 100	20	0.874
Career Satisfaction Briscoe et al. (2021), Achievement	71	12.70	72	76	20, 100	20	0.916
Objective Career Success % Salary Increase	13.55	26.41	10	0	−100, 100	1	—
Objective Career Success No. of Promotions	0.94	1.263	1	0	0, 10	1	—

*Perceived self-efficacy* (Kossek et al., 1998): 11 items (e.g., “*Please indicate the extent to which you agree or disagree with each of the following statements: “When I make plans for my career, I am confident I can make them work; If I cannot do a job the first time, I keep trying until I can*”), using a 5-point scale (1 = strongly disagree, to 5 = strongly agree). Some items were reverse-coded so that higher perceived self-efficacy was always represented by higher values.

*Desire for career control* (King, 2000): 7 items (e.g., “*Please indicate how important it is for you to have control over: which employer you work for; The hours you work*”), using a 5-point Likert-type scale (1 = Not at all important, to 5 = Extremely important).

*Perceived organizational support* (Eisenberger et al., 1986): 11 items (e.g., “*Please indicate the extent to which you agree or disagree with each of the following statements: the organisation values my contribution to its well-being; the organisation fails to appreciate any extra effort from me*”), using a 5-point Likert-type scale (1 = Strongly disagree, to 5 = Strongly agree). Some items were reverse-coded so that higher perceived organizational support was always represented by higher values.

*Strategic career behaviours* (*Kaleidoscope Career Model*, Sullivan and Mainiero, 2008): 15 items, 5 each for the authenticity, balance, and challenge subscales (e.g., “*Please indicate the extent to which each of the following statements describes you: I hunger for greater spiritual growth in my life; I constantly arrange my work around my family needs; I continually look for new challenges in everything I do*”), using a 5-point Likert-type scale (1 = This does not describe me at all, to 5 = This describes me very well).

*Perceived career control* (Kuijpers and Scheerens, 2006): 5 items (e.g., “*Please indicate the extent to which you agree or disagree with each of the following statements: I can make clear career plans; I know what I want to have achieved in my career a year from now*”), using a 5-point Likert-type scale (1 = Strongly disagree, to 5 = Strongly agree).

*Subjective career success* (Briscoe et al., 2021): level of importance of 20 items measured on a 5-point Likert-type scale (1 = Not at all important, to 5 = Extremely important), (e.g., “*Please indicate the importance to you of: having the opportunity to be innovative in my work activities; Experiencing challenges in my work; Continuously learning throughout my career*,” etc.).

*Career satisfaction* (Briscoe et al., 2021): level of achievement on the same 20 items as above (e.g., “*In regard to this career aspect, I have achieved a level I am happy with*…”), measured on a 5-point Likert-type scale (1 = Strongly disagree, to 5 = Strongly agree).

*Objective career success* (Whitely et al., 1991): measured using percentage of salary increase and number of promotions received in the previous 6 years.

### Data collection and data analysis procedures

This study is part of a wider project funded through Fundação para a Ciência e Tecnologia (FCT; Foundation for Science and Technology), I.P. under the EXPL/PSI-GER/0321/2021 project – EURECA: new career strategies for new European remote careers. This was reviewed and approved by the Católica Research Centre for Psychological, Family and Social Wellbeing (CRC-W) Review Board. The assessment protocol, consisting of the previously mentioned instruments, was implemented online on the Qualtrics platform, and later imported to the Prolific platform. This later platform allows access to a database of potential participants, according to criteria defined by the researchers: (i) to be over 18 years old, (ii) to be a resident in a European country, and (iii) to work remotely. Participants were informed of all necessary ethical procedures through informed consent. The following definition of remote work was also provided: remote work (or telework/telecommute) is a work arrangement in which employees work from home or from another remote location, via the internet, email, or phone, instead of commuting to a central office. Participants received a small financial compensation for the time spent (£2; approximately €2.34). Data were collected in June 2022 and analysed using SPSS (IBM, Version 28).

Pearson correlation analysis was conducted between all factors in the model. Linear regression analysis was used to test: Perceived Self-Efficacy, Desire for Career Control, and Perceived Organizational Support as predictors of Strategic Career Behaviours; Strategic Career Behaviours as a predictor of Perceived Career Control, Subjective Career Success, and Career Satisfaction; as well as Perceived Self-Efficacy as a predictor of Subjective Career Success; Perceived Organizational Support as a predictor of Perceived Self-Efficacy and Career Satisfaction; Perceived Career Control as a predictor of Career Satisfaction; and Subjective Career Success as a predictor of Perceived Career Control and Career Satisfaction. Mediation Analysis, using PROCESS model 4 (Hayes, 2013), was used to test the mediating effects of Strategic Career Behaviours between the antecedent and consequent factors.

## Results

Results from Pearson correlation analysis (Table 2) indicated that the measures of Objective Career Success (OCS) had only weak correlations with the other variables and were hence removed from the model.

**Table 2:** Correlations between antecedents and consequences of strategic career behaviours.

a. Correlations *p < 0.05, **p < 0.01, and ***p < 0.001								
Variable	1	2	3	4	5	6	7	8
1. PSE	––							
2. DCC	0.254***	––						
3. POS	0.228***	0.052	––					
4. SCB	0.348***	0.437***	0.120**	––				
5. PCC	0.541***	0.251***	0.258***	0.435***	––			
6. SCS	0.355***	0.593***	0.092*	0.587***	0.355***	––		
7. SAT	0.366***	0.256***	0.307***	0.361***	0.458***	0.428***	––	
8. OCS (%SI)	0.092*	0.085*	0.097*	0.033	0.057	0.073	0.108**	––
9. OCS (PRO)	0.106**	0.029	0.085*	0.053	0.135***	0.059	0.158***	0.224***

Strong correlations were found between most other variables, except Desire for Career Control and Perceived Organizational Support which was insignificant, and Perceived Organizational Support and Subjective Career Success, which was significant (*p* < 0.05), but mild (*r* = 0.092). The direction of the correlation between Perceived Self-Efficacy and Desire for Career Control was positive, in contrast to the negative correlation found in the preliminary study. The number of significant correlations was also greater than in the preliminary study, allowing for further investigation into the predictive and mediating effects of Strategic Career Behaviour and the relationships between the antecedents and consequences.

Direct regression analyses (Table 3) were conducted between correlated factors.

**Table 3:** Regression results between antecedents and consequences of strategic career behaviours.

Regressions: direct paths								
IV	DV	B	SE (B)	*β*	*R*^2^	*R*^2^ adj.	*t*	*p*
PSE	SCB (path a)	0.511	0.051	0.348	0.121	0.120	14.823	<0.001
––	DCC (path e)	0.130	0.018	0.254	0.065	0.063	7.139	<0.001
––	PCC	0.340	0.019	0.541	0.293	0.292	17.479	<0.001
––	SCS (path f)	0.555	0.054	0.355	0.126	0.125	10.322	<0.001
––	SAT	0.740	0.069	0.366	0.134	0.133	10.675	<0.001
DCC	SCB (path b)	1.261	0.096	0.437	0.191	0.190	13.179	<0.001
––	PCC	0.310	0.044	0.251	0.063	0.062	7.048	<0.001
––	SCS	1.817	0.091	0.593	0.351	0.351	19.983	<0.001
––	SAT	1.016	0.141	0.256	0.065	0.064	7.183	<0.001
POS	SCB (path c)	0.218	0.066	0.120	0.014	0.013	3.287	0.001
––	PSE (path d)	0.282	0.044	0.228	0.052	0.051	6.362	<0.001
––	PCC	0.200	0.028	0.258	0.066	0.065	7.235	<0.001
––	SCS	0.178	0.071	0.092	0.008	0.007	2.512	0.012
––	SAT (path g)	0.766	0.088	0.307	0.094	0.093	8.745	<0.001
SCB	PCC (path h)	0.186	0.014	0.435	0.189	0.188	13.111	<0.001
––	SCS (path j)	0.623	0.032	0.587	0.345	0.344	19.692	<0.001
––	SAT (path k)	0.496	0.047	0.361	0.130	0.129	10.495	<0.001
PCC	SAT (path l)	1.474	0.105	0.458	0.210	0.209	13.981	<0.001
SCS	PCC (path m)	0.143	0.014	0.355	0.126	0.125	10.299	<0.001
––	SAT (path n)	0.554	0.043	0.428	0.183	0.182	12.845	<0.001

All regression paths were significant, including those not expected in the model, and all at the *p* < 0.001 level, with the exception of Perceived Organizational Support as a predictor of Strategic Career Behaviours (*p* = 0.001) and of Subjective Career Success (*p* = 0.012).

When combined, the antecedents explained 25.3% of the variance in Strategic Career Behaviours (Table 4). In this model, perceived self-efficacy and desire for career control were significant predictors of strategic career behaviours, but perceived organizational support was not.

**Table 4:** Regression results between antecedents and strategic career behaviours.

a. Outcome: strategic career behaviours (SCB)							
*R*	*R*^2^	MSE	*F*	df1	df2	*p*	
0.503	0.253	8.00	82.791	3	735	<0.001	
Main model		Coeff (B)	SE	*t*	*p*	LLCI	ULCI
PSE (path a^)		0.357	0.050	7.173	<0.001	0.259	0.454
DCC (path b^)		1.076	0.095	11.302	<0.001	0.889	1.263
POS (path c^)		0.083	0.059	1.388	0.166	−0.034	0.199

*n* = 738; confidence for all CIs in output: 95.0000. No of bootstrap samples for percentile bootstrap confidence intervals: 5000.

The results from subsequent mediation analysis using PROCESS model 4 (Hayes, 2013) tested the degree to which Strategic Career Behaviours mediated the effects of the antecedents on the consequences (Table 5).

**Table 5:** Mediation results between antecedents and consequences of strategic career behaviours.

Mediation										
a. Outcome: perceived career control (PCC; Blue paths, Figure 2)										
*R*	*R*^2^	MSE	*F*	df1	df2	*p*				
0.615	0.379	3.12	111.789	4	734	<0.001	Indirect effects on PCC			
Main model	Coeff (B)	SE	*T*	*p*	LLCI	ULCI	Effect	SE	LLCI	ULCI
PSE (path o)	0.260	0.020	12.953	<0.001	0.220	0.299	0.0613	0.0097	0.0432	0.0813
DCC (path r)	0.029	0.040	0.722	0.470	−0.050	0.108	0.2164	0.0278	0.1647	0.2745
POS (path u)	0.101	0.023	4.347	<0.001	0.055	0.146	0.0382	0.0126	0.0146	0.0642
SCB (path h^)	0.113	0.014	7.879	<0.001	0.142	0.435				

Mediation										
b. Outcome: subjective career success (SCS; Red paths, Figure 2)										
*R*	*R* ^2^	MSE	*F*	df1	df2	*p*				
0.760	0.498	6.96	182.186	4	734	<0.001	Indirect effects on SCS			
Main model	Coeff (B)	SE	*T*	*p*	LLCI	ULCI	Effect	SE	LLCI	ULCI
PSE (path p)	0.196	0.045	4.385	<0.001	0.108	0.284	0.2862	0.0035	0.2210	0.3530
DCC (path s)	1.225	0.090	13.647	<0.001	1.049	1.401	0.5429	0.0569	0.4378	0.6588
POS (path x)	−0.003	0.052	−0.064	0.949	−0.105	0.098	0.1354	0.0443	0.0496	0.2241
SCB (path j^)	0.392	0.032	12.203	<0.001	0.329	0.455				

Mediation										
c. Outcome: career satisfaction (SAT; Green paths, Figure 2)										
*R*	*R*^2^	MSE	*F*	df1	df2	*p*				
0.501	0.251	11.027	61.356	4	734	<0.001	Indirect effects on SAT			
Main model	Coeff (B)	SE	*T*	*p*	LLCI	ULCI	Effect	SE	LLCI	ULCI
PSE (path q)	0.434	0.071	6.122	<0.001	0.295	0.573	0.1866	0.0321	0.1264	0.2519
DCC (path t)	0.375	0.142	2.638	0.009	0.096	0.654	0.5333	0.0802	0.3849	0.7005
POS (path z)	0.566	0.082	6.895	<0.001	0.405	0.727	0.0986	0.0342	0.0328	0.1685
SCB (path k^)	0.299	0.051	5.884	<0.001	0.199	0.399				

*n* = 738; confidence for all CIs in output: 95.0000. No of bootstrap samples for percentile bootstrap confidence intervals: 5000.

When combined, the antecedents and Strategic Career Behaviours significantly predicted all three consequences. In predicting Perceived Career Control, Desire for Career Control was no longer a significant predictor but did have significant indirect effects, and Perceived Self-Efficacy and Perceived Organizational Support remained significant predictors and had significant indirect effects. This suggests that the effect of Desire for Career Control on Perceived Career Control is significantly mediated by Strategic Career Behaviours, but that the effects of Perceived Self-Efficacy and Perceived Organizational Support on Perceived Career Control are only partially mediated by Strategic Career Behaviours.

In predicting Subjective Career Success, Perceived Organizational Support was no longer a significant predictor but did have significant indirect effects, and Perceived Self-Efficacy and Desire for Career Control remained significant predictors and had significant indirect effects, suggesting that the mild effect of Perceived Organizational Support on Subjective Career Success is significantly mediated by Strategic Career Behaviours, but that the effects of Perceived Self-Efficacy and Desire for Career Control on Subjective Career Success are only partially mediated by Strategic Career Behaviours.

In predicting Career Satisfaction, all three antecedents remained significant predictors, however there was a drop in the significance of Desire for Career Control as a predictor (*p* < 0.001 to *p* = 0.009). This suggests that the effects of the antecedents on Career Satisfaction are only partially mediated by Strategic Career Behaviours, with a greater partial mediation for Desire for Career Control.

**Figure 2:**
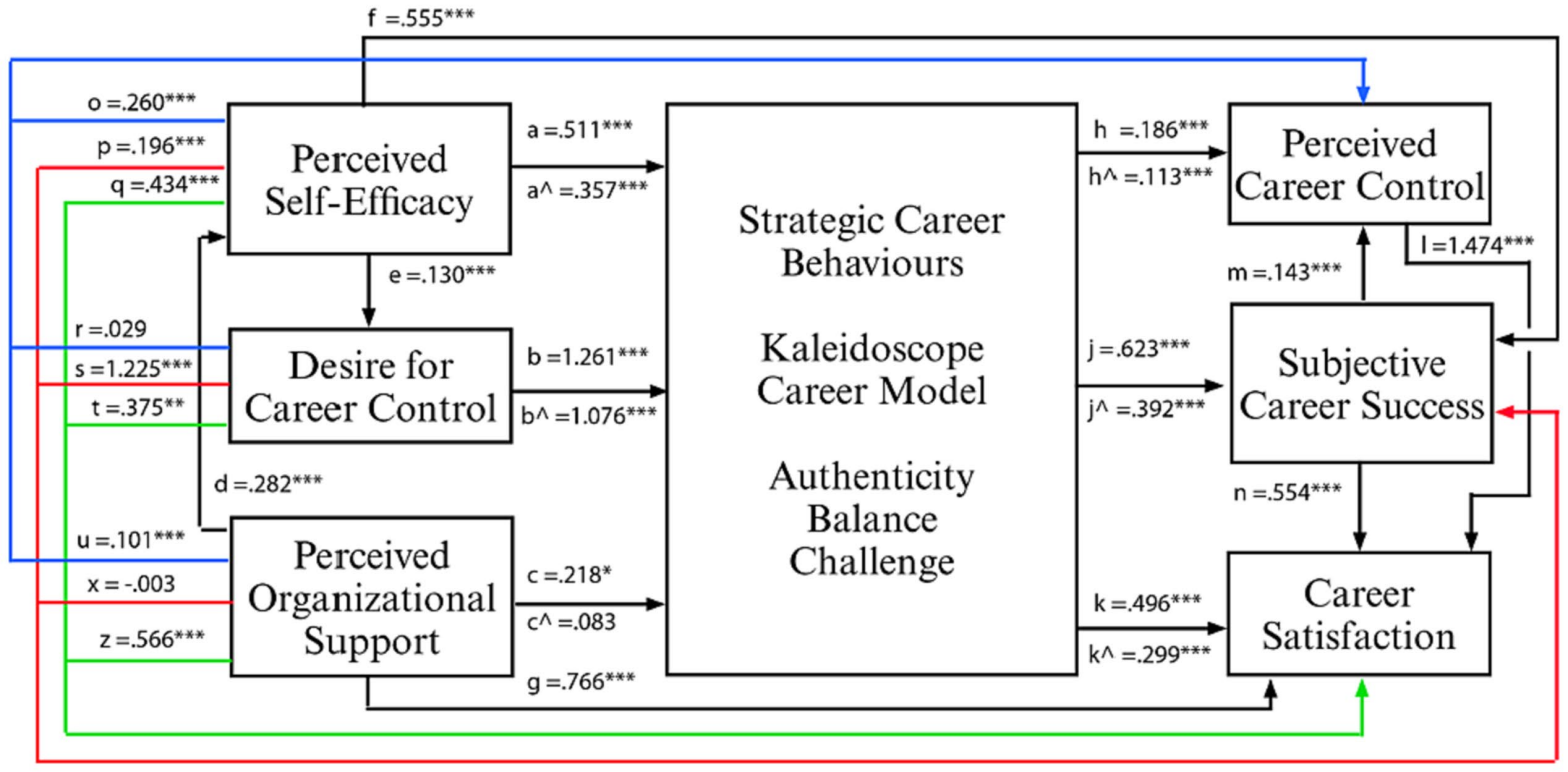
Analysed unstandardized coefficients of the career management model. *p < 0.05, **p < 0.01, and ***p < 0.001; coloured lines: indirect effect of each antecedent variable on perceived career control (blue), subjective career success (red), and career satisfaction (green) when the relationships are mediated by strategic career behaviours.

A completed model of the effect sizes is displayed in Figure 2 below.

## Discussion

The aim of the present study was to analyse the antecedents and consequences of strategic career behaviours among a sample of European remote workers. The regression paths between all variables, including those not expected in the model, were found to be significant within the *p* < 0.05 level (most at the *p* < 0.001 level).

Regarding the relationships between the antecedents, and between them and Strategic Career Behaviours, Perceived Self-Efficacy positively predicted Desire for Career Control, this is in contrast to *H1e*, but aligns with the arguments of King (2000, 2004) that increases in perceived self-efficacy occur when the act of exercising career control produces positive outcomes that drive the desire for more career control. Interestingly, the correlation between Perceived Self-Efficacy and Desire for Career Control in the present study was positive, as was the predictive value of Perceived Self-Efficacy on Strategic Career Behaviours (in contrast to *H1a*), both of which are in the opposite direction to that found in the preliminary study. A negative relationship between Perceived Self-Efficacy and Desire for Career Control [as found by Sotto-Mayor (2022) and Sotto-Mayor et al. (2023)] makes sense if feeling competent and efficacious translates to a sense career control, hence reducing any desire to gain control, whereas a positive relationship [as found by King (2004)] would suggest that feeling efficacious leads one to have a strong desire for career control because they believe they have the capability to gain that control.

Similarly, a negative relationship between Perceived Self-Efficacy and Strategic Career Behaviours [as shown by Sotto-Mayor et al. (2023)] would suggest that already feeling competent and efficacious reduces the need to engage in behaviours to develop those competencies, whereas a positive relationship [as shown by Raghuram et al. (2003)] would suggest that feeling efficacious leads one to seek out career strategies in order to make use of those competencies.

Furthermore, Desire for Career Control positively predicted Strategic Career Behaviours (supporting *H1b*), which would suggest that such a desire leads individuals to find strategic ways to realise that control, as supported by Sotto-Mayor et al. (2023) and King (2004). This would also suggest a potential partially mediating, or at least complimentary, role for desire for career control in the positive relationship between self-efficacy and strategic career behaviour.

However, it is possible that there are two cases for those individuals with a strong desire for career control: desiring career control because the individual does not have it; or, already having career control but desiring to keep it or have more of it. While the positive relationship between Desire for Career Control and Perceived Career Control (contrary to *H3b*) suggests that the majority of the present sample represents the latter case, Desire for Career Control scored low on the reliability measure (Cronbach’s *α* = 0.552), which may be due to these two cases presenting differently when surveyed with the measure for Desire for Career Control used in this study. Either way, further analysis would be needed to tease these two cases apart and to determine whether such dynamics are at play.

Lastly, Perceived Organisational Support positively predicted Perceived Self-Efficacy, supporting *H1d*. This would suggest that a feeling of support from an organisation is an important factor in developing a positive view of one’s capabilities. For hybrid workers, this may be even more important, as a greater sense of self-efficacy is required to overcome the challenging tasks posed by the remote/at-home environment (Kossek et al., 1998; King, 2000; Raabe et al., 2007; Van Vianen et al., 2008). Receiving support from one’s organisation is also important for well-being (Desrosiers, 2001), which would account for the effect Perceived Organizational Support had on Career Satisfaction in the present results (in partial support of *H3c*). However, the mediation analysis showed that when the antecedents were combined, the model was a significant predictor of Strategic Career Behaviours, but only Perceived Self-Efficacy and Desire for Career Control continued to have significant direct or indirect effects, whereas Perceived Organizational Support did not contribute significantly. This may be due to the role agency plays in the variables. Of the three antecedents, Perceived Organizational Support would be expected to be more reliant on the variability of organisations and the actual support those organisations give, and less on the variability of individual characteristics. These results support *H1c* and, because Perceived Organizational Support was a significant predictor of Perceived Self-Efficacy, suggest that the latter may mediate the mild direct effect of Perceived Organizational Support on Strategic Career Behaviours. This would make sense if receiving or perceiving support from one’s organisation helps bolster the feelings of self-efficacy that lead to engagement in strategic career behaviours and career outcomes, as partially evidenced by Liu et al. (2015), but is not necessary for it.

The present findings suggest that the sample is most representative of individuals for whom feeling supported by their organisation plays a small role in their sense of self-efficacy, which in turn leads to a desire for career control because self-efficacy represents a belief in their capability to gain that control, and which they seek to gain through strategic career behaviours. However, considering the contrary findings in the preliminary study, these present findings may represent only a small majority of the sample. Segmentation of the sample may elucidate potential differences in the underlying mechanisms leading to strategic career behaviours, as well as other moderating or mediating factors.

Regarding the relationships between Strategic Career Behaviours and the consequences, as well as the direct relationships between the antecedents and consequences, the variable Strategic Career Behaviours was a positive predictor of Perceived Career Control, Subjective Career Success and Career Satisfaction, fully supporting *H2a*, with the exception of objective career success measures being removed from the model. This suggest that engaging in strategic career behaviours is a key factor in feelings of career control, success and satisfaction. Therefore, engaging in strategic career behaviours involves adopting a proactive and intentional approach to career development and management (Mainiero and Sullivan, 2005; Sullivan and Mainiero, 2008). It entails taking responsibility for one’s own professional progress and taking consistent steps (e.g., setting clear professional goals, seeking learning and growth opportunities, building a strong professional network, seeking feedback to improve skills), to achieve desired career goals, which enhances the sense of career control (King, 2000, 2004; Pinto, 2010) over the direction and development of the career, i.e., the individual believes they have the capacity to influence outcomes and make decisions that positively impact their professional trajectory, (ii) increases the attainment of successful outcomes, such as career advancement, achievement of professional goals, recognition by peers and the organization, among other indicators of success, and (iii) results in greater job satisfaction because individuals feel more in control of their own professional destiny and are working towards goals that are meaningful to them (King, 2004; Greenhaus et al., 2010; Pinto, 2010; Green et al., 2020). In summary, taking active and strategic measures regarding one’s career can help individuals feel more in control, achieve success, and experience greater job satisfaction.

Desire for Career Control was a positive predictor of Perceived Career Control, contradicting the no direct effects hypothesised in *H3b*. Furthermore, when the antecedents were combined with Strategic Career Behaviours to predict Perceived Career Control, Desire for Career Control no longer had a direct effect, but still had significant indirect effects, suggesting that Strategic Career Behaviours mediates the relationship, providing partial support to *H4*. This would make sense if either having a desire for career control stems from a lack of it, as suggested above and as would be suggested by McDonald and Hite’s (2008) findings, or if in order to simultaneously desire control and feel as though one has it requires being engaged in strategic career behaviours, as the results from the previous study by Sotto-Mayor et al. (2023) suggested, aligning with King (2004).

Perceived Self-Efficacy and Perceived Organizational Support also positively predicted Perceived Career Control, although no relationships were hypothesised. They also remained significant predictors of Perceived Career Control in the presence of Strategic Career Behaviours while also having significant indirect effects, suggesting that Strategic Career Behaviours partially mediates the relationships, and providing additional partial support to *H4*. This combination of direct and indirect effects would make sense if having a strong sense of one’s capabilities, as well as having a firm organisational base of support to work from both directly bolster a sense of career control as well as motivating the engagement in strategic career behaviours which further adds to that sense of control, as suggested by Abdalla (1995), Nabi (2000), and Taylor and Popma (1990), and indirectly suggested by the findings of Agrawal and Singh (2022).

Perceived Self-Efficacy and Desire for Career Control were both positive predictors of Subjective Career Success (supporting *H3a* but contrary to *H3b*). Additionally, when the antecedents were combined with Strategic Career Behaviours to predict Subjective Career Success, both Perceived Self-Efficacy and Desire for Career Control had significant indirect effects but remained significant direct predictors. This suggests that both a strong sense of one’s capabilities and a desire for career control can alone provide a sense of career success as well as motivate engagement in the strategic career behaviours that help one achieve that success. This follows findings from previous studies (Tannenbaum et al., 1991; King, 2000) showing an effect of self-efficacy on job performance and motivation, which in turn can affect Subjective Career Success (Heslin, 2005), and these relationships also follow findings by Abele and Spurk (2009), Badri et al. (2013), Ballout (2009), King (2004), Kuijpers and Scheerens (2006), Raghuram et al. (2003), Rigotti et al. (2020), Sotto-Mayor et al. (2023), and Wu et al. (2022). Both sets of result suggest a partial mediation of those relationships by Strategic Career Behaviours, adding support to *H4*. These studies highlight the importance of self-efficacy in professional development, effective leadership, school-to-work transition, and workplace well-being, demonstrating how individual beliefs about their own abilities can influence behavior and performance. All of these studies indicate that beliefs about one’s abilities influence the confidence needed to explore career options, make informed decisions, and tackle professional challenges and opportunities.

Perceived Organizational Support was a positive predictor of Subjective Career Success (supporting *H3c*), but only a very weak one (*R*^2^ = 0.008, *p* = 0.012). Furthermore, the mediation analysis showed that when the antecedents were combined with Strategic Career Behaviours to predict Subjective Career Success, Perceived Organizational Support no longer had a direct effect, but still had significant indirect effects, suggesting that Strategic Career Behaviours fully mediates this relationship (providing partial support for *H4*). The fact that Perceived Organizational Support is also not a significant predictor of Strategic Career Behaviours in the presence of the other antecedents suggests the very weak indirect effect of Perceived Organizational Support on Subjective Career Success may be further mediated by Perceived Self-Efficacy, as proposed above. These results suggest that organizational support may not play an important role in determining whether, either because tools are available so that strategic behaviours can be engaged in with or without the support of the organisation, or because the present sample represents highly independent individuals. Indeed, such independence may be expected more from those able to sustain a sense of career control and career satisfaction while working remotely. However, further analysis would be needed to investigate these differences.

Perceived Self-Efficacy, Perceived Organisational Support and Desire for Career Control were all positive predictors of Career Satisfaction (supporting *H3a* and *H3c* but contrary to *H3b*). However, Perceived Self-Efficacy was not as a predictor as it had been in the prior study of Sotto-Mayor et al. (2023), which may be due to the use of an alternative measure of career satisfaction (Greenhaus et al., 1990 vs. Briscoe et al., 2021 [achievement]). Combined with Strategic Career Behaviours to predict Career Satisfaction, all three remained significant predictors, but still had significant indirect effects, suggesting only partial mediation of these relationships by Strategic Career Behaviours. This suggests that a sense of satisfaction in one’s career requires a sense of self-efficacy, support from one’s organisation and a desire to be in control of one’s career and requires or is aided by the engagement in strategic career behaviours, taking advantage of those supports, desires and capabilities. The first two and the last relationships have been suggested by Abele and Spurk (2009), Badri et al. (2013), Ballout (2009), and Rigotti et al. (2020); Agrawal and Singh (2022) and Armstrong-Stassen and Ursel (2009); and Greenhaus et al. (1990), Lau and Pang (2000), and Raabe et al. (2007), respectively, though no support for the relationship with Desire for Career Control. This lack of support may be hinted at in the results by the slight drop in the significance of Desire for Career Control as a direct predictor of Career Satisfaction, potentially suggesting that in order for a desire for career control to contribute to career satisfaction, that desire needs to lead to strategic career behaviours, however further analysis would be needed to determine this speculation. Overall, the mediation analyses provided partial and varied support for *H4*.

In the direct relationships between the consequences, Perceived Career Control was a strong positive predictor of Career Satisfaction (supporting *H2b*), accounting for 21% of the variance. This finding points to the importance a sense of career control has in feeling satisfied with that career, at least among the present European sample. Similarly to previous studies (King, 2000, 2004), this result underscores the importance of encouraging the development of a sense of autonomy and control in one’s career, for example, through programs and interventions in this area, which can significantly contribute to career satisfaction and well-being.

Subjective Career Success was a positive predictor of both Perceived Career Control and Career Satisfaction (fully supporting *H2d*), This means that individuals who perceive themselves as successful in their careers are more likely to feel they have control over their careers and experience satisfaction. However, given the strength of the relationship between Perceived Career Control and Career Satisfaction, the causal relationship between Subjective Career Success and Perceived Career Control may be from the latter to the former. In other words, feeling in control of one’s career can shape perceptions of success by influencing how individuals attribute their achievements, fostering positive self-evaluation and resilience, and promoting alignment with personal values and aspirations. This highlights the importance of perceived career control in shaping individuals’ overall satisfaction and well-being in their professional lives.

Support for *H2c* was indeterminate due to the removal of Objective Career Success measures from the model.

Many of the correlation and regression relationships in the present study had not been found in the preliminary study (Sotto-Mayor et al., 2023). This greater number of correlations suggests that a larger and more diverse sample provided a more nuanced overview of career behaviours and outcomes which may not have been present due to idiosyncrasies of the Iberian labour market or of the small sample (*N* = 96) drawn from this region in the previous study.

The fact that Strategic Career Behaviours were found to play only a partial mediating role in the present model suggest that further analysis is required to determine whether they play a central role in the relationships between the antecedents and consequences in the present model, or whether they should be considered a contributing but merely parallel factor.

Further investigation is needed to determine other mediating or moderating variables in the complex interactions between the antecedents and consequences of strategic career behaviours.

## Limitations

### Sample

Responses were received from residents of 17 different countries across Europe. However, a third of those were from Portugal (31.9%), a quarter from Poland (26.0%), and another quarter from Italy, Spain and Greece combined (26.4%). That equates to 58.3% of respondents residing in southern European countries, and only 15.7% from northern European countries other than Poland, with only Hungary (*N* = 32, 4.3%) and the United Kingdom (*N* = 35, 4.7%) individually contributing more than 1.5%. The partial skew in favour of southern European countries, and the potentially unrepresentative numbers from most northern European countries, suggest that findings may be characteristic of southern European countries.

Of those that responded to questions regarding salary (*N* = 686), most (76.2%, *N* = 523) reported salaries below 1,500€, and reported earning average (57.0%) or below average (39.8%) wages compared to their colleagues. This presents two issues: if the sample is only representative of low-salary countries, that may affect the engagement in strategic career behaviours; and, if the sample is representative of individuals with low wages, regardless of country averages, this may indicate a self-selection bias when respondents are reimbursed for completing the questionnaire. Respondents received £2.51 (~2.89€) for valid completion of the questionnaire, which took on average 15 min to complete. The effective value of this reimbursement would differ for those earning different wages. Indeed, the sample may be representative of lower-than-average incomes, with the average upper bounds of monthly salaries being 1,600€, well below average for Europe (37,500€ *per annum*; EUROSTAT, 2022).

Most respondents were unmarried (67.5%, *N* = 499) or had no children (86.5%, *N* = 639), and the average age was 27.64 years, despite the range extending from 18 years to 70. This may represent a self-selection bias, as those young, unmarried or without children have more time to complete a questionnaire, in comparison to those who must dedicate considerable time their relationship, to raising children, or to both.

Thus, in the future, it will be necessary to continue collecting new participant samples with greater diversity in terms of their sociodemographic characteristics (e.g., age, marital status, family composition, geographic location). This is aimed at creating a career management model that is truly representative and generalizable to the population of European remote workers.

### Measures of objective career success

The measures for Objective Career Success were self-reported number of promotions received and percentage increase in salary within the last 6 years. The scale for increase in salary ranged from −100 to +100%, which restricted the capacity for some possibilities to be accurately reported. While a salary cannot be reduced by more than 100%, it can be increased by more than 100%. An individual working part-time while studying and making 1,000€ per month, and then graduating into a salary of 4,000€ per month would see a 300% increase in their salary. Restricting the scale to +100% did not allow for such instances to be accurately reported. Furthermore, the self-report nature of the measure required respondents to make a roundabout calculation of the percentage increase, which may have led to a large variability in the accuracy of the response and hence may help explain, along with the other factors, why Objective Career Success failed to correlate with the other variables in the model when operationalised in this way. This situation has also been observed in previous studies (e.g., Sotto-Mayor et al., 2023), highlighting the need to consider alternative measures of objective career success. For future studies, it is suggested to directly inquire about salary and bonus progression over time, assess not only the number of promotions but also the significance of advancements in job title or responsibilities, as well as awards, accolades, or recognition from peers, employers, and industry associations.

## Conclusion

Hybrid and remote working have grown in prevalence since the onset of the COVID-19 pandemic, and there are many unique challenges face by workers, especially those working from home, in developing and managing their careers. Hence there is new-found reason to investigate the career management strategies and outcomes of those participating in this new way of working and to support those individuals in managing their careers. The current state of the literature investigating interactions between the variables in our model have been so far mostly pairwise, and only a portion of those are focused on remote workers, and an even smaller portion on European remote workers.

The present research aimed to extend the work by Sotto-Mayor et al. (2023) and analyse their model of antecedents and consequences of strategic career behaviours among teleworkers across Europe.

The results showed that personal factors such as perceived self-efficacy and a desire for career control are important determiners of workers engaging in strategic career behaviours. Engaging in these behaviours is in turn important for achieving control, success and satisfaction in one’s career. The results also showed that feeling supported by the organisation you work for improves the chances of feeling in control of your career, as well as more satisfied with that career.

The results also suggest that by encouraging employees to take control of their careers and by helping them to gain the tools that lead them to feel a greater sense of self-efficacy, supervisors and human resource managers can indirectly improve the sense of control, success and satisfaction their employees have in their careers, which may also have immeasurable positive effects on the culture and wellbeing of the organisation.

Luckily for supervisors and human resources managers, they can imbue a greater sense of career control and satisfaction in their employees simply by making them feel supported by the organisation they work for. This could be achieved by offering opportunities for employees to improve their skills in a supportive and collaborative environment, or by improving the internal communication channels within the organisation so that employees can voice issues and participate in the decision-making that affects them.

The present research will go towards developing additional recommendations and career management strategies for organisations and individuals engaged in hybrid and remote working across the European continent, while accounting for the nuanced differences in the goals and beliefs of individuals that can affect their career outcomes.

## Data availability statement

The raw data supporting the conclusions of this article will be made available by the authors, without undue reservation.

## Ethics statement

The studies involving humans were approved by Católica Research Centre for Psychological, Family and Social Wellbeing (CRC-W) Review Board. The studies were conducted in accordance with the local legislation and institutional requirements. The participants provided their written informed consent to participate in this study.

## Author contributions

KH: Formal analysis, Investigation, Methodology, Writing – original draft, Writing – review & editing. MP: Supervision, Validation, Writing – review & editing. SC: Supervision, Validation, Writing – review & editing. JP: Conceptualization, Data curation, Funding acquisition, Project administration, Supervision, Validation, Writing – review & editing.

## Footnotes

^1^ https://ucpcienciashumanas.eu.qualtrics.com/jfe/form/SV_egQUJXlNwiGwQPs
